# Application of a new designed high resolution melting analysis for mycobacterial species identification

**DOI:** 10.1186/s12866-024-03361-x

**Published:** 2024-06-08

**Authors:** Azar Dokht Khosravi, Hossein Meghdadi, Mohammad Hashemzadeh, Ameneh Alami, Mohammad Reza Tabandeh

**Affiliations:** 1 Infectious and Tropical Diseases Research Center, Health Research Institute, Ahvaz Jundishapur University of Medical Sciences, Ahvaz, Iran; 2 Department of Microbiology, Faculty of Medicine, Ahvaz Jundishapur University of Medical Sciences, Ahvaz, Iran; 3 Department of Basic Sciences, Division of Biochemistry and Molecular Biology, Faculty of Veterinary Medicine, Shahid Chamran University of Ahvaz, Ahvaz, Iran

**Keywords:** Multilocus sequence analysis, High Resolution Melting, *Tuf*, *atpE*, *rpoB*, *dnaK*

## Abstract

The Non-tuberculous mycobacterial (NTM) isolates should be distinguished from tuberculosis and identified at the species level for choosing an appropriate treatment plan. In this study, two molecular methods were used to differentiate NTM species, including a new designed High Resolution Melting (HRM) and Multilocus Sequence Analysis (MLSA). Seventy-five mycobacterial isolates were evaluated by sequencing four genes ( MLSA) and a HRM assay specifically targeting *atpE* was designed to rapidly and accurately identify and differentiate *mycobacterium* species. Out of 70 NTM isolates, 66 (94.3%), 65 (92.9%), 65 (92.9%) and 64 (91.4%) isolates were identified to the species level by PCR of *atpE, tuf*, *rpoB and dnaK* genes. We could identify 100% of the isolates to the species level (14 different species) by MLSA. By using HRM assay, all NTM isolates were identified and classified into eight groups, in addition, Mycobacterium tuberculosis and Nocardia were also detected simultaneously. The MLSA technique was able to differentiate all 14 species of NTM isolates. According to the results, the HRM assay is a rapid and beneficial method for identifying NTM, *M. tuberculosis* (MTB), and Nocardia isolates without sequencing.

## Background

The genus Mycobacterium consists of more than 200 species that are divided into three main groups [1], including *Mycobacterium tuberculosis* complex (MTBC), *Mycobacterium leprae*, and non-tuberculous mycobacteria (NTM). *M. tuberculosis* (MTB) is still a significant concern worldwide despite the many advances in diagnostic techniques and treatment. According to the World Health Organization (WHO), approximately 9.9 million people become sick with TB globally, equivalent to 127 cases per 100,000 population in 2020 [2]. On the other hand, NTM infections have also grown significantly worldwide and have become essential pathogens [3]. These ubiquitous organisms can cause infections in various body sites such as pulmonary disease, skin and soft tissue infections, lymphadenitis, bone infection, disseminated disease, and otitis media [4–6]. As MTBC can also invade multiple organs of the body [7], therefore, differentiating MTBC from NTM is very important because the treatment and medication regimen and their drug sensitivity are different, even within the closely related species of NTM [8–10]. According to the American Thoracic Society (ATS) guideline, the NTM isolates obtained from clinical specimens should be identified at the species level for patients treatment management [11].

Identifying NTM isolates at the species level using traditional methods based on culture and biochemical tests is cumbersome, time-consuming, and usually leads to ambiguous results [12]. It seems unavoidable to use molecular tests such as PCR with gene sequencing for a more specific and reliable diagnosis of Mycobacteria [13]. Recent studies show that performing PCR-sequencing with a single target causes non-differentiation of closely related species [14–16].

Therefore, using several housekeeping genes and Multilocus Sequence Analysis (MLSA) method based on concatenated sequences to diagnose *Mycobacterium* species is one of the ways to overcome discrimination limitations [17–19]. The high-resolution curve melting (HRM) assay is a method for identifying Mycobacterial species. The assay follows Real-Time PCR in a single tube and is based on analyzing fluorescence curves produced by labeled dye binding to double-stranded DNA during strand dissociation events in the melting phase. This method is used to identify single nucleotide polymorphisms (SNPs), genotyping, nucleic acid methylation, and species identification [20–22]. In this study, we evaluated Real-Time PCR-HRM assay for identification and differentiation of different mycobacterial species, MLSA (concatenate the four genes) was used as the gold standard for molecular diagnosis of Mycobacterial species.

## Materials and methods

In total eighty isolates including seventy-five mycobacterial isolates (*M. kansasii*, *M. fortuitum*, *M. simiae*, *M. avium*, *M. abscessus, M. porcinum, M. paraintracellular, M. intracellular, M. yongonense, M.gordonae, M.paragordonae, M.pulveris, M. conceptionense, M. lentiflavum*, *M. tuberculosis* complex ) and five clinical isolates of *N. nova* were selected from the archive of samples collected from patients referred to the selected TB Reference Centres of Tehran and Ahvaz, Iran, in a 2 year period from April 2021 to April 2023. The preliminary proposal of the study was approved by the Institutional Ethics and Review Board (IR.AJUMS.REC.1399.033) of the Ahvaz Jundishapur University of Medical Sciences, Iran. All isolates were cultured on the Löwenstein-Jensen (LJ) medium [23]. The DNA of the colonies was extracted by a High Pure PCR Template Preparation Kit (Roche-Germany), according to the manufacturer’s recommendations. The extracted DNA was stored at -20 °C until PCR amplification. The reference strains of N. nova CCUG 70,657, M. tuberculosis H37Rv, M. fortuitum ATCC 49404T, M. abscessus ATCC 23,003, M. avium ATCC 25,291, and M. kansasii ATCC 12478T were used as control strains in all stages of the study.

### Multiple sequence alignment

All 80 isolates were evaluated by PCR targeting four different genes of *rpoB* [24], *tuf* [25], *dnaK* [26], and *atpE* [27] was performed to identify all isolates to species level using the primers listed in Table 1. The final volume of DNA amplification reaction for individual genes targets was 25 µl and consisted of 10X PCR buffer, 1.5 mM MgCl_2_, 10 mM dNTPs, 0.5 µM of each primer, 1.5 U of Super Taq™ DNA polymerase (Roche, Germany), and 2 µl of template DNA. The PCR program was performed as below: initial denaturation at 95 °C for 5 min, followed by 32 cycles of denaturation at 95 °C for 45 s, annealing of specific primers at an appropriate temperature (Table 1) for 45 s, extension at 72 °C for 1 min, and final extension at 72 °C for 5 min. The PCR products were separated by electrophoresis on a 2% agarose gel.

**Table 1:** Primers used for PCR

gene	Primers	Product size	Anealing temp
*rpoB*	F:5’-GGCAAGGTCACCCCGAAGGG-3′ R:5’-AGCGGCTGCTGGGTGATCATC-3′	723 bp	59ºC
*tuf*	T1 5’-CACGCCGACTACATCAAGAA-3’ T2 5’-GAACTGCGGACGGTAGTTGT-3’	652 bp	48 ºC
*dnaK*	dnaKF1: 5’-CTGACCAAGGACAAGATGGC-3′ dnaKR1: 5’-TCGATCAGCTTGGTCATCAC-3′	451 bp	56ºC
*atpE*	FatpE 5′-CGGYGCCGGTATCGGYGA-3′ RatpE 5′-CGAAGACGAACARSGCCAT-3′	182 bp	58 ºC

The amplified fragments of *rpoB*, *tuf*, *dnaK*, and *atpE* genes were sequenced and analyzed in GenBank (http://www.ncbi.nlm.nih.gov/BLAST/) to initial association with reference strains. The sequences related to each gene entered into the jPhydit program separately and aligned with standard NTM strain sequences. The aligned nucleotide sequences of *rpoB*, *tuf*, *dnaK*, and *atpE* genes were concatenated and analyzed by this software and MEGA (Molecular Evolutionary Genetics Analysis) software version 6.0 [28, 29].

### Optimization of real-time PCR and high-resolution curve melting

Real-Time PCR was carried out using *atpE* primers [27] listed in Table 1. The PCR reaction was performed using Type-it HRM PCR Kit (QIAGEN, Germany). Each PCR reaction was prepared in a total volume of 25 µl that included: a 2X master mix (12.5 µl), each primer (0.7 µl), RNase-free water (9.1 µl), and template DNA (2 µl). Real-time PCR and HRM were performed using a Rotor-Gene 6000 (QIAGEN, Germany). The PCR for generating amplicons for HRM analysis was performed using the following conditions: an activation step at 94 °C for 5 min, 40 cycles of denaturation at 95 °C for 10 s, annealing at 55 °C for 30 s, and extension at 72 °C for 10 s. The HRM analysis follows as temperature increases from 80 °C to 95 °C at a rate of 0.1 °C per step increments every 2 s, with persistent fluorescence detection. The post-PCR HRM curve analysis was carried out using Rotor-Gene 6000 Series Software 1.7.

## Results

PCR results revealed that all mycobacterium isolates were positive for *rpoB*, *tuf*, *dnaK*, and *atpE* targets. The sequences of all four genes were entered into the jPhydit program separately after being aligned with standard strain sequences. For MLSA analysis, all four aligned nucleotide sequences of genes were concatenated and entered into the jPhydit software after aligning with standard strain sequences. The results obtained from PCR of genes *rpoB*, *tuf*, *dnaK*, and *atpE* and as well as MLSA results are listed in Table 2.

**Table 2:** identification of clinical isolates

isolates	Identification by									
	rpoB		dnaK		tuf		atpE		MLSA	
	D	M	D	M	D	M	D	M	D	M
*M. kansasii*	5	0	5	0	5	0	5	0	5	0
*M. fortuitum*	5	0	5	0	5	0	5	0	5	0
*M. simiae*	5	0	5	0	5	0	5	0	5	0
*M. avium*	5	0	5	0	5	0	5	0	5	0
*M. abscessus*	5	0	5	0	5	0	5	0	5	0
*M. porcinum*	5	0	5	0	5	0	5	0	5	0
*M. paraintracellulare*	5	0	4	1	5	0	2	3	5	0
*M. yongonense*	5	0	3	2	2	3	5	0	5	0
*M. intracellulare*	3	2	5	0	5	0	5	0	5	0
*M. paragordonae*	5	0	4	1	5	0	5	0	5	0
*M. gordonae*	4	1	4	1	3	2	5	0	5	0
*M. lentiflavum*	5	0	5	0	5	0	5	0	5	0
*M. conceptionense*	5	0	5	0	5	0	4	1	5	0
*M.pulveris*	3	2	4	1	5	0	5	0	5	0
*M. tuberculosis*	5	0	5	0	5	0	5	0	5	0
*N. nova*	5	0	5	0	5	0	5	0	5	0
***Total***	**75**	**5**	**74**	**6**	**75**	**5**	**76**	**4**	**80**	**0**

D: differentiated to the species level

M: Not differentiated to the species level

**Fig. 1:**
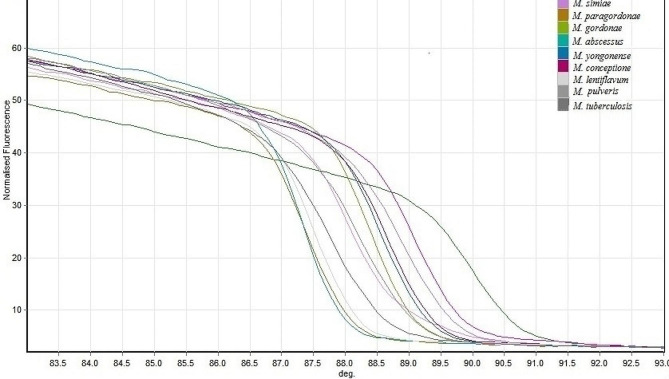
Normalized melting curves of *atpE*, illustrating the high-resolution melting (HRM) for identification of *Mycobacterium* isolates and *N. nova* isolate

The HRM assay in this study was designed by a fragment of 182 bp of the *atpE* gene to identify 80 clinical isolates. Each isolate showed a specific melting temperature and plot pattern. *Mycobacterium* isolates are differentiated by more than ± 0.2 relative fluorescence unit (RFU) cut-offs, while isolates with less than ± 0.2 RFU were classified into one group. According to these criteria, isolates were classified into 10 groups. *M. abscessus* and *M. paragordonae* with 87.3 °C were placed together in group (I) *M. lentiflavum with* 87.5 °C was placed in group (II) *M. kansasii*, *M. fortuitum*, *M. avium* with 88.0 ± 0.1 °C were placed group (III) *M.pulveris* with 88.2 °C was placed in group (IV) *M. gordonae, M. yongonense, M. intracellulare*, and *M. paraintracellulare* with 88.4 ± 0.1 °C were placed together in group (V) *M. conceptionense* with 88.6 °C was placed in group (VI) *M. simiae* with 88.8 °C was placed in group (VII) *M. porcinum* with 89.0 °C was placed in group (VIII) *M. tuberculosis* with 87.8 °C was placed in group (IX) *N. nova* with 90.0 °C was placed in group X (Fig. 1).

## Discussion

Identifying and differentiating of NTM species from MTBC members is crucial because most NTM isolates are inherently resistant to anti-TB drugs and even though, the duration of treatment for NTM is also different. Differentiation between these two groups are difficult by traditional methods, therefore, in areas where TB is endemic, the NTM infections are misdiagnosed as TB and the agent is reported as MTB [30, 31]. According to the ATS guideline, the clinical NTM isolates should be identified at the species level for choosing an appropriate treatment plan [11]. Appropriate and rapid molecular diagnostic methods for the identification of mycobacterial species should be developed to avoid unnecessary treatment and delay in appropriate treatment regimen.

In this study, four genes tuf, *rpoB*, *dnaK* and *atpE* were used as targets in PCR-sequencing method. The three genes *tuf*, *rpoB*, and *dnaK* were introduced as the highest power to detect and distinguish *Mycobacterium* species in previous studies (24,25,26), and the *atpE* gene was used in this study because it was introduced as a new identification tool (27). seventy NTM isolates were identified by *rpoB*, *dnaK, tuf*, and *atpE* genes using the PCR-sequencing method. Sixty five out of 70 NTM isolates were well identified by the *rpoB* gene, and 5 (7.1%) isolates included 2 (2.9%) *M. intracellulare*, 1(1.4%) *M. gordonae* and 2 (2.9%) *M .pulveris* weren’t well differentiated. Sixty four isolates were well identified by the *dnaK* gene, but 1 (1.4%) *M. paraintracellulare*, 2 (2.9%) *M. yongonense*,1(1.4%) *M. paragordonae*, 1 (1.4%) *M. gordonae*, and 1 (1.4%) *M .pulveris* weren’twell differentiated. Sixty five out of all NTM isolates were well identified by the *tuf* gene, and 3 (4.28%) *M. yongonense* and 2 (2.9%) *M. gordonae* weren’t well differentiated. The atpE gene was able to differentiated 66 (94.3%) of 70 NTM isolates from each other correctly but this fragment couldn’t correctly distinguish 3 (4.28%) *M. paraintracellulare* and 1 (1.4%) *M. conceptionense*. *N. nova* and *M. tuberculosis* complex isolates were identified by 4 genes.

The aligned nucleotide sequences of *rpoB*, *tuf*, *atpE*, and *dnaK* were concatenated to increase the identification and differentiation of clinical mycobacteria. Based on the maximum similarity, 14 species (100% isolates) were identified. In another study, Kim et al. used 3 PCR targets, *16Sr RNA*, *hsp65* and *rpoB gene*s and improved their results by 97.3% using MLSA [32]. The difference between the results and our study may be due to differences in the isolates studied and used fewer targets. Hashemi et al. used four genes, including *16SrRNA*, *rpoB, hsp65* genes, and ITS region, and could identify all species by MLSA (18). Similar to our study, they were able to distinguish all species. MLSA has also been shown to successfully identify clinical mycobacterium isolates in other studies [29, 33].

In this study, the HRM assay was designed by an *atpE* fragment to identify 70 *NTM* isolates. All NTM isolates were classified into eight HRM groups. Each group showed a specific melting temperature from 87.3 to 89.0 °C. In this method, *M. tuberculosis* with 87.8 °C melting temperature was placed separately in group IX. Also, *N. nova* could be identified and differentiated from *Mycobacterium* species by this method, and with 90.0 °C was placed in group X.

Discrimination of closely related species of *Mycobacterium* by traditional culture and phenotypic methods is very tedious and ambiguous. On the other hand, PCR-Sequencing based on single target also does not have the ability to completely differentiate the species from each other. Due to its very high identification and differentiation of clinical mycobacteria, the MLSA technique has recently been recognized as the molecular standard in the diagnosis of non-tuberculous mycobacteria, but due to its long process including multi-gene PCR, and sequencing of individual genes, building a concatenated chain of several genes and its analysis are practically only used in the research field. But the HRM assay to identify mycobacterium species has clinical application. The high speed and proper accuracy of this method distinguish many species in less than 3 h. One of the advantages of HRM assay is the ability to separate NTM from TB simultaneously, which is very useful and vital. A number of clinical specimens referred to specialized TB centers are actually specimen from Nocardia infection because clinical and radiological findings are not specialized. On the other hand, laboratory tests including microscopic smears and culture may be wrongly identified as mycobacterium. Some patients are even mistakenly treated with anti-mycobacterial drugs and are interpreted as drug-resistant tuberculosis due to lack of proper treatment [34]. Therefore, proper diagnosis of *Nocardia* from Mycobacterium is very important. Simultaneous differentiation of *Nocardia* from *Mycobacterium* species is another extraordinary advantage of this method, which quickly identifies the species causing the infection and determines the treatment line of the disease. The low price of real-time PCR-HRM assay compared to other methods like probe-based real-time PCR is a great advantage. On the other hand, in this method, unlike probe-based real-time PCR, there is no need to design a specific probe and primer for each species separately.

The real-time PCR method has been used to detect and identify *Mycobacterium* isolates, which can identify a maximum of three to four species in a reaction tube [35–37]. In this study, we designed a real-time PCR-HRM assay, which could successfully identify the clinical mycobacterial species, including *M. tuberculosis, M. fortuitum, M. kansasii*, *M. simiae*, *M. avium*, *M. abscessus, M. yongonense, M. intracellulare, M. paraintracellulare, M. gordonae, M. porcinum, M. paragordonae, M. lentiflavum, M. conceptionense* and *M. pulveris* in 9 HRM group. Issa et al. evaluated the HRM assay using *16 S rRNA* as the target gene for differentiation of *Mycobacterium* isolates. However, they could not identify some common species such as *M. fortuitum*, *M. kansasii*, *M. simiae*, and *M. abscessus* [21]. Chen et al. developed a dual-target real-time PCR-HRM assay by combined *hsp65* and *16 S rRNA* target, and they were able to identify a large number of NTM species in 12 HRM groups. The results of their study were as good as ours; however, they used dual-target, and we targeted a single gene [20]. Perng et al. evaluated the real-time PCR-HRM assay targeting the *16 S rRNA* gene and ITS region to detect mycobacterial isolates. 101 isolates out of 134 isolates were divided into four groups, including *M. chelonae* group, M. *gordonae* group, *M. avium* group, and *M. fortuitum* group; in comparison to our study, they could identify fewer distinct groups [38].

## Conclusion

In conclusion, this study proved that the most reliable method for detecting and differentiating mycobacterium isolates from each other is the MLSA method, which can differentiate 100% between species. Since this method is based on sequencing and needs to construct a concatenated sequence and analyze it, MLSA is a difficult method. In real-time PCR-HRM assay, there is no need for gene sequencing and all the analysis is done in one tube, so this method very fast and quickly detects the Mycobacterium species and determines the treatment line.

## Data Availability

All analyzed data within this study can be obtained from the corresponding author on request.
